# Enrichment of circulating melanoma cells (CMCs) using negative selection from patients with metastatic melanoma

**DOI:** 10.18632/oncotarget.1683

**Published:** 2014-02-03

**Authors:** Powrnima Joshi, Barbara Jacobs, Adeeb Derakhshan, Lee R. Moore, Paul Elson, Pierre L. Triozzi, Ernest Borden, Maciej Zborowski

**Affiliations:** ^1^ Department of Biomedical Engineering, Lerner Research Institute, Cleveland Clinic, 9500 Euclid Avenue, Cleveland, OH; ^2^ Taussig Cancer Institute, Cleveland Clinic, 9500 Euclid Avenue, Cleveland, OH; ^3^ Cleveland Clinic Lerner College of Medicine of Case Western Reserve University, 9500 Euclid Avenue, Cleveland, OH; ^4^ Quantitative Health Sciences, Cleveland Clinic, 9500 Euclid Avenue, Cleveland, OH

**Keywords:** circulating melanoma cells, CTC, Melan-A, S100B, magnetic separation, negative selection

## Abstract

Circulating tumor cells have emerged as prognostic biomarkers in the treatment of metastatic cancers of epithelial origins *viz*., breast, colorectal and prostate. These tumors express Epithelial Cell Adhesion Molecule (EpCAM) on their cell surface which is used as an antigen for immunoaffinity capture. However, EpCAM capture technologies are of limited utility for non-epithelial cancers such as melanoma.

We report a method to enrich Circulating Melanoma Cells (CMCs) that does not presuppose malignant cell characteristics. CMCs were enriched by centrifugation of blood samples from healthy (N = 10) and patient (N = 11) donors, followed by RBC lysis and immunomagnetic depletion of CD45-positive leukocytes in a specialized magnetic separator. CMCs were identified by immunocytochemistry using Melan-A or S100B as melanoma markers and enumerated using automated microscopy image analyses. Separation was optimized for maximum sensitivity and recovery of CMCs.

Our results indicate large number of CMCs in Stage IV melanoma patients. Analysis of survival suggested a trend toward decreased survival with increased number of CMCs. Moreover, melanoma-associated miRs were found to be higher in CMC-enriched fractions in two patients when compared with the unseparated samples, validating this method as applicable for molecular analyses.

Negative selection is a promising approach for isolation of CMCs and other EpCAM -negative CTCs, and is amenable to molecular analysis of CMCs. Further studies are required to validate its efficacy at capturing specific circulating cells for genomic analysis, and xenograft studies.

## INTRODUCTION

Biomarkers for monitoring onset of cancer, disease progression, and response to treatment have a large impact on clinical management and outcomes. In the past decade Circulating Tumor Cells (CTCs) have been investigated as potential biomarkers. CTCs are cells dislodged from the primary tumor, invading endothelium, lymph, and peripheral blood, reaching distant sites to initiate and promote metastasis. Numerous studies with metastatic breast, prostate and colorectal cancers have validated CTCs as adverse prognostic markers for disease-free and overall survival. These studies have identified the change in number of CTCs in patient blood as predictive of treatment or therapy outcomes [1-5]. The presence of CTCs in cerebrospinal fluid (CSF) has also been validated for breast cancer patients with brain metastasis, and can serve potentially as an early marker for the involvement of brain as a secondary site [6]. Other biomarkers such as circulating tumor DNA (ctDNA) and mutant mitochondrial DNA (mtDNA) are being investigated for potential insights into genomic stability, heterogeneity, cancer progression, and tailoring patient specific treatment options (personalized medicine) [7-9].

Breast, prostate and colorectal cancers of epithelial origin carry a shared cell surface marker known as Epithelial Cell Adhesion Molecule or EpCAM. Immunomagnetic antibodies against EpCAM have been used to target the CTCs, followed by magnetic separation and optical analysis to isolate and reliably detect CTCs (CellSearch® Circulating Tumor Cell Test, Janssen Diagnostics, LLC, Raritan, NJ) [3, 10]. This method of using an antigen expressed by the tumor cells as means of their capture and isolation is referred to as “positive selection”. However, most circulating melanoma cells (CMCs) do not express EpCAM. Melanoma is the malignant transformation of melanocytes of neural crest origin and has been associated with the cell surface marker CD146 and Melanoma-associated Chondroitin Sulphate Proteoglycan (MCSP). Indeed, CD146 has been proposed as a substitute for EpCAM in immunomagnetic targeting and separation of melanoma cells using positive selection [11].

An alternative to the “positive selection” CTC capture strategy is “negative selection” in which cells of interest are enriched by depletion of unwanted cells. Negative selection is advantageous for separating cells with poorly characterized immunophenotype. Importantly, the enriched cells are “untouched” by the labeling ligands, and therefore, less likely to be activated. The CTCs thus captured offer an unbiased representation of their biology. Both these attributes are highly desirable for probing questions in cancer biology and validating biomarkers.

We have developed a negative cell separation strategy that relies on a combination of viscous flow and magnetic force that facilitates recovery of the unlabeled cells (CTCs) from whole blood samples obtained from cancer patients [12-15]. The process consists of removal of normal blood cells from the patient blood sample by centrifugation and erythrocyte lysis followed by pan-leukocyte marker (CD45) antibody labeling of leukocytes and their depletion by magnetic separation. The magnetic separator consists of a cylindrical flow channel placed in a quadrupole magnet (Supplementary Figure S1). The radially-oriented magnetic force drives the magnetically tagged leukocytes towards the channel wall (the cell fraction termed Retentate); the viscous flow shear stress of the axially-oriented fluid flow, on the other hand, minimizes the non-specific trapping of the non-magnetic cells on the channel wall and facilitates their elution from the magnet (the cell fraction termed Eluate). Separation is achieved in a matter of minutes (less than 15 minutes for a sample containing 10^8^ cells) with an enrichment of up to 10,000-fold [14]. In principle, the unlabeled cell fraction in Eluate is enriched in CTCs and is available for analysis by immunocytochemistry, real time qPCR, fluorescence *in-situ* hybridization (FISH), genomic analysis and microarray based techniques, as well as xenografting studies.

In the present study we have adapted the negative cell separation strategy for the analysis of CMCs from blood samples donated by patients with metastatic melanoma. The method provided us with a convenient platform to test combinations of different antibodies and fluorochrome tags for CMC identification by immunocytochemistry and high resolution fluorescence microscopy. Healthy donor blood was used as a negative control. Enrichment of circulating, tumor specific microRNA in the CMC enriched fraction (Eluate) as compared to the unseparated sample was measured as an additional validation of the efficiency of separation process, similarly to isolation of mRNAs from melanoma CTCs by other methods [16]. This study focused on the application of Melan-A and S100B as CMC markers, in combination with CD45 as a leukocyte marker, and a semi-automated computer image analysis protocol, for high specificity of the CMC detection.

## RESULTS

### Melanoma markers (Melan-A and S100B) show specificity and heterogeneity in staining with SKMEL-28 melanoma cell line spiked into normal blood

**Figure 1:**
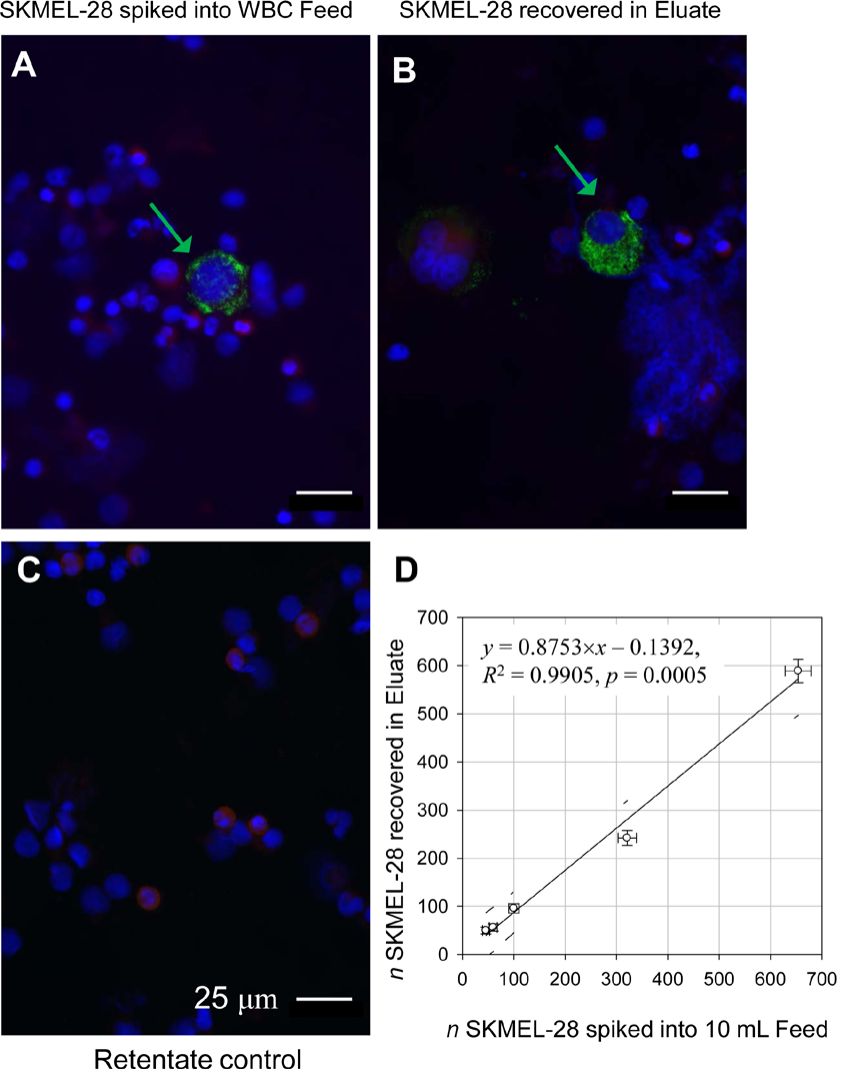
Melan-A positive and CD45 negative cells correlate with the SKMEL-28 melanoma cells added to the leukocyte preparation White blood cell fraction (“buffy coat”) from 10 mL of healthy donor blood spiked with SKMEL-28 melanoma cell line and separated into two fractions by magnetically tagging with anti-CD45 antibody and magnetic flow sorting. Cells are stained with antibodies against Melan-A (Green) and CD45 (Red). The cell nuclei are visualized by DAPI staining (Blue). (A) Original sample (Feed). (B) Non-magnetic Eluate fraction enriched for spiked SKMEL-28 cells. (C) Magnetic Retentate fraction rich in leukocytes retained on magnet. Note the depletion of leukocytes in the Eluate fraction (B) compared with the Feed (A) and Retentate (C); note also the differences in Melan-A positive staining between cells indicating Melan-A antigen heterogeneity (A and B, Green arrows). Scale bar = 25 μm. (D) Linear regression (solid line) shows that the spiked cells are fully recovered in the Eluate fraction (within 95% confidence interval).

S100B and Melan-A proteins are present in melanocytes and most melanomas [17]. We therefore, chose these proteins as markers to detect CMCs. Antibodies against S100B and Melan-A were tested individually in cell line spiking experiments using SKMEL-28 cells to optimize the staining process. A pan-leukocyte CD45 antibody, targeted to a different epitope than the antibody used for magnetic leukocyte depletion, was used as a negative counterstain to increase specificity of CMC identification by immunocytochemistry. The resulting images of Melan-A positive cells stained in green color (Figure 1A,B arrows) and the leukocytes stained in red color, showed no apparent cross reactivity of the melanoma markers with the leukocytes (Figure 1A,B). There was a noticeable heterogeneity in staining for melanoma biomarkers in SKMEL-28 cells, where some cells stained brighter than others (green arrows, Figure 1A, B). Similar results occurred with antibody to S100B (data not shown). Note also that the cultured SKMEL-28 cells were visibly larger than the leukocytes (red arrows).

### SKMEL-28 cells were recovered with high efficiency from spiked blood following negative selection

The magnetic leukocyte depletion resulted in marked decrease of CD45 positive red-stained cells (leukocytes) in the Eluate fraction thus enriching the spiked SKMEL-28 cells in that fraction (as compared to the original sample, Feed, Figure 1 A,B, respectively). As expected, the Retentate contained most of the leukocytes (Figure 1C). To determine the limit of detection (LOD) of the negative separation method, the spiked SKMEL-28 cells captured in the Eluate were counted and plotted against the number of SKMEL-28 cells added to the blood buffy coat sample before sorting. When assessed by regression analysis, the limit of sensitivity of detection was 10 SKMEL-28 cells per 1 mL blood (Figure 1D). The limit was independent of the cell line, as confirmed by adding 100 cultured OCM1a uveal melanoma cells to 10 mL normal whole blood and repeating the experiment. The depletion rate of the leukocytes in the separator was between 98-99% (data not shown) comparable to what we have reported previously [14]. Similar to SKMEL-28 cells, the cultured OCM1a cells were larger than the normal blood leukocytes (Supplementary Figure S3).

### CMCs were detected robustly in blood from metastatic melanoma patients

Work described above on assessment of patients' blood resulted in development of criteria to define a CMC (Supplementary Table S1). The effort was on developing criteria that minimized operator bias and took into account expected patient-to-patient variability. Both CMC inclusion and exclusion criteria are used (namely, three positive identification rules and three negative identification rules, Supplementary Table S1). For instance, the presence of a nucleus is a necessary but not sufficient condition for cell identification as the nucleus margin needs to be well defined for an imaged object to be included in the cell count.

**Figure 2:**
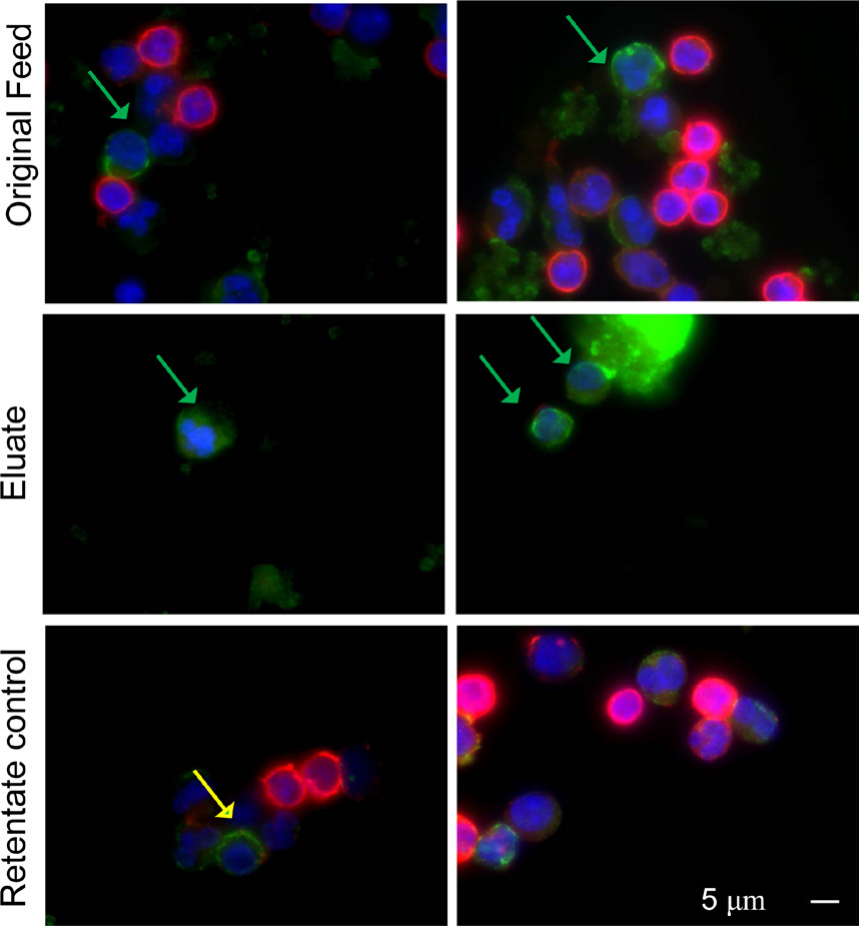
Negative selection method increases frequency of CMCs in the depleted blood cell fractions Cells from patient blood sample before (Feed) and after negative cell separation (Eluate and Retentate) followed by immunocytochemistry visualization. Blue - DAPI (cell nuclei), Red – CD45 (leukocytes), Green – S100B. Green arrows show putative CMCs. Yellow arrows show dual staining on a cell, perhaps due to melanoma antigens being present in the leukocytic subpopulations by phagocytosis. Scale bar = 5 μm.

Large size of the cells was not deemed to be a necessary CMC attribute *a priori*. Specifically, there was a significant difference in cell sizes between the melanoma cell cultures and cells positive for CMC markers found in patients' blood (compare Supplementary Figure S3 and Figures 1A and B with Figure 2, respectively). SKMEL-28, OCM1a and other established melanoma cell lines had large cells easily discernible using hematoxylin and eosin stain, or Wright-Giemsa stain (Supplementary Figure S3) and by immunocytochemistry using Melan-A staining (Figure 2). However, with patient samples all cells were similar to each other with no clear size distinction between cells positive or negative for melanoma markers (either Melan A or S100B) (Figure 2). Typically, melanoma cells produced uniform or punctate cytoplasmic staining pattern for Melan-A (Figure 2) and both cytoplasmic and nuclear distribution for S100B. This characteristic staining pattern was used as one of the CMC selection criterion (Supplementary Table S1).

Care was taken to eliminate confounding effects of cellular debris. For the automated method, the false positive counts often resulted from dense debris concentration on the slide (Supplementary Figure 5A) requiring subsequent manual elimination from the counts. Cells positive for both CD45 and melanoma markers were not counted as CMCs. The identity of these dual positive cells is unknown, and we have considered but not tested the possibility of these cells being of hematopoietic origin capable of presenting the tumor antigens. Cells were screened for both Melan-A and S100. Cells that expressed the highest amount of either Melan-A or S100B were quantified as CMCs.

Blood samples from 10 normal donors processed using the same protocol as that developed for patient samples resulted in 0-3 CMC/mL (Figure 4 and Supplementary Table S2). Overall, the normal samples produced images with low background staining indicating high specificity of the Melan-A and S100B antibody (Supplementary Figure S6).

**Figure 3:**
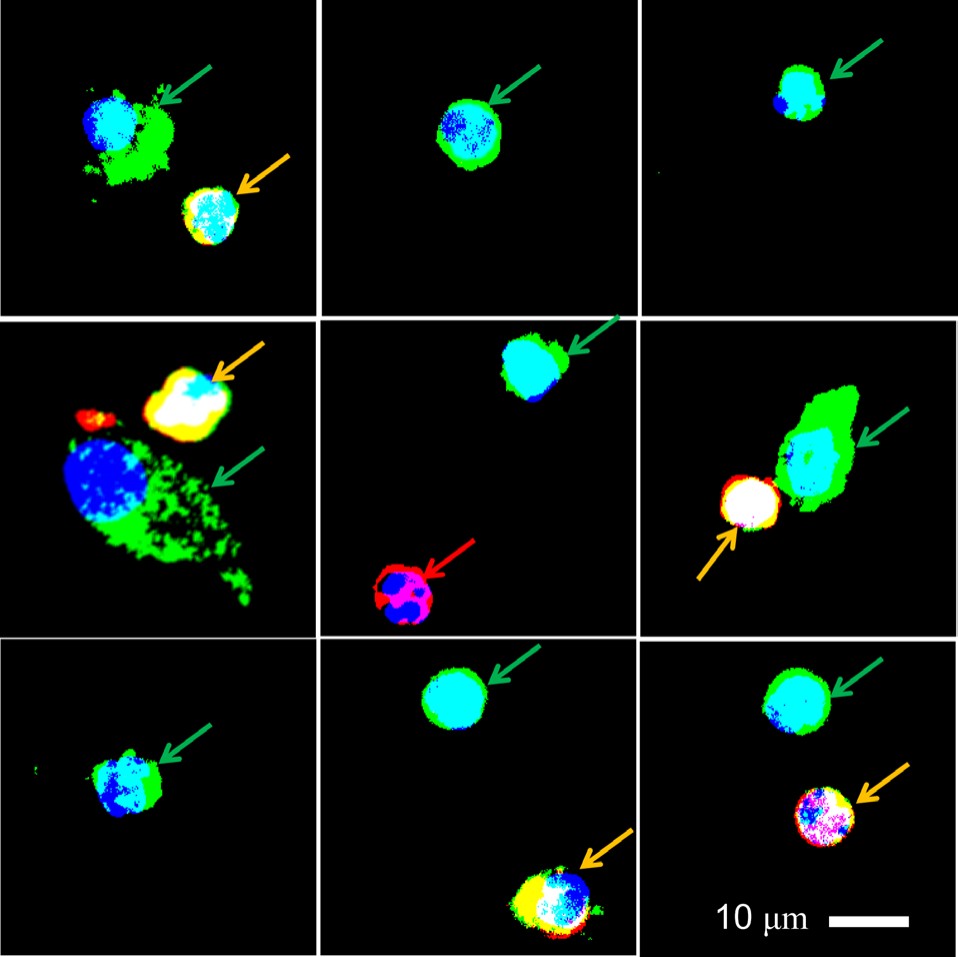
Gallery of CMCs isolated from 6 patients Note the difference in size and morphology between CMCs (Green arrows). Some are as small as leukocytes (Red arrow), while others larger or somewhat elongated. Yellow arrows indicate dual positive cells that may be leukocytes expressing the tumor antigens.

**Figure 4:**
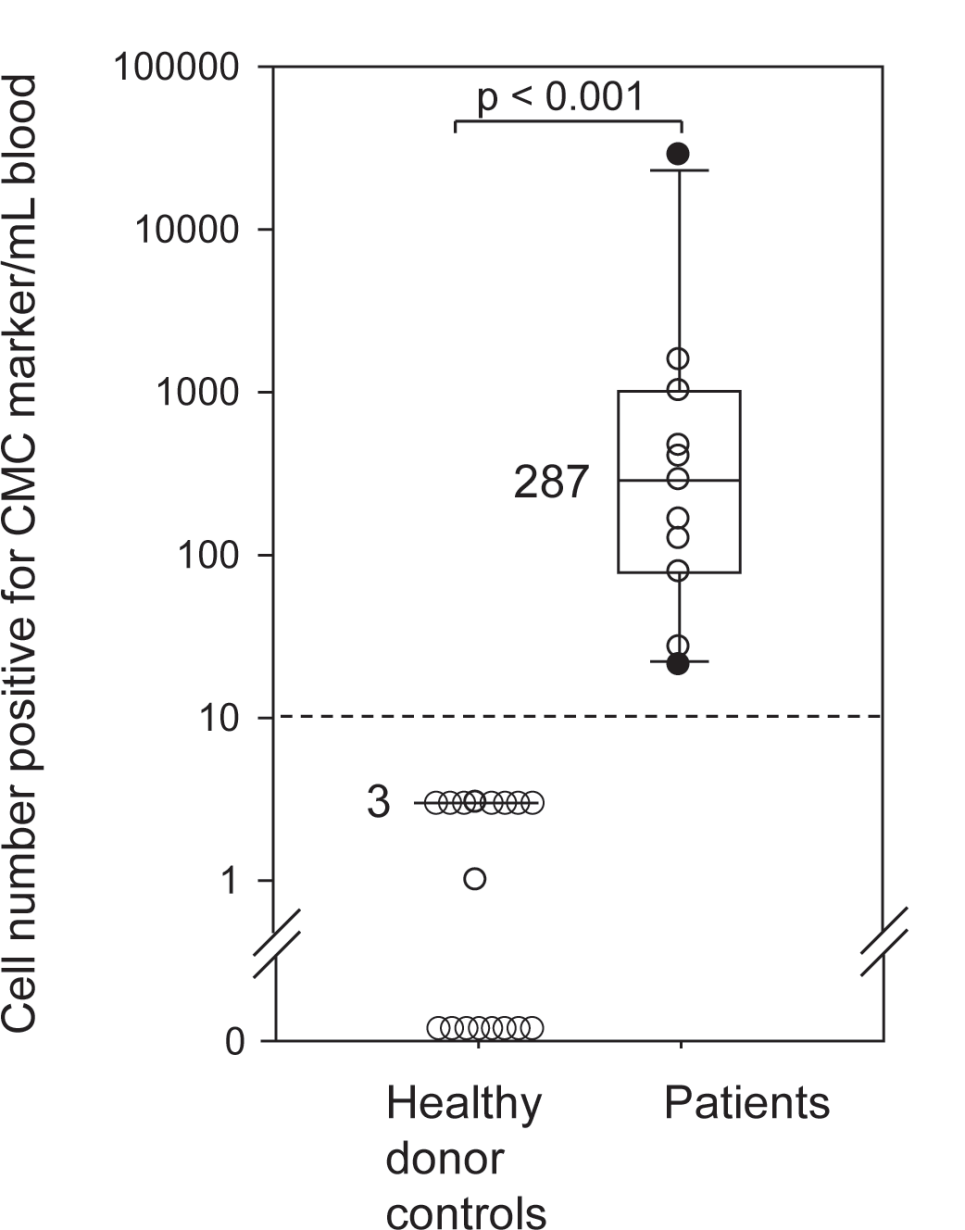
CMC numbers in Stage IV melanoma patients are significantly higher than the positives detected in normal blood by the negative selection method Comparison between healthy donor blood samples (N = 10 for two markers combined) and patient blood samples (N = 11) processed and analyzed for CMCs. Box plots show median and quartiles. The broken line indicates limit of detection determined by spiking experiments. The two groups are statistically different (p <0.0001 using Mann-Whitney two tailed test).

### Blood samples from metastatic melanoma patients show large number of CMCs

The median CMC count for all (N = 11) patient samples was 287 CMCs/mL of whole blood as compared to <3 positive cells /mL in healthy donors. (p < 0.001; Table 1 and Figure 4). The results were broadly distributed for CMC numbers in patients with metastatic Stage IV melanoma with a range of 21 to over 28,000 per mL of whole blood (Table 1). In comparison, the normal samples (N=10) showed 0 to 3 positive cells per mL of blood (Figure 4). The high CMC numbers suggested high sensitivity for CMC enrichment and detection considering the no, or occasional low, positive count in healthy controls. CMC numbers were an order of magnitude higher than those reported using other devices or studies with other types of cancer [10, 11, 18, 19].

**Table 1:** Patient demographics and survival in the order of increasing CMC counts

No.	Survival in Months	nCMC/slide	CV%	nCMC/mL	BRAF V600E mutation
1	5.5^b^	4	50	21	Positive
2	4.6^b^	10	32	27	Positive
3	31.9^b^	79	11	78^c^	Positive
4^a^	3.0	47	15	125	Positive
5^a^	2.0	31	18	165^c^	Positive
6	27.8	101	10	287	Negative
7	0.5	75	12	400	Not determined
8^a^	33.2^b^	42	15	467^c^	Not determined
9	28.9^b^	304	5.7	1,013^c^	Positive
10^a^	4.5	1,646	2.5	1,568	Not determined
11^a^	1.0	2,134	2.2	28,483	Positive

a Males. Ten patients were diagnosed with Stage IV cutaneous melanoma. Three patients, No. 7, 8 and 10 were diagnosed with Stage IV, uveal and mucosal melanomas respectively. The patient age ranged from 49 to 74 yr. CV is the relative standard deviation (coefficient of variation) estimated from Poisson distribution using nCMC/slide as a mode.

b Alive at the time of analysis.

c Four patients were tested with S100B; the rest of the patients were tested with Melan-A.

### CMC counts: correlation trend with patient survival

The wide dynamic range of the CMC counts allowed us to perform an overall survival analysis based on these patients with Stage IV metastatic melanoma (Table 1). Patient characteristics were the following: 5 males (45%), 6 females (55%); median age at the time of sample was 63 years (range 49-74); Sites: cutaneous N = 8 (73%), mucosal N = 2 (18%); uveal N = 1 (9%); median CMC 287 (range 21-28,483). BRAF: (+) N = 7 (88%, all 7 had cutaneous melanoma), (-) N = 1 (12%), unknown N = 3 (Table 1). Five patients out of 11 were alive at the time of analysis. Four out of the 11 patients had CMCs reported with S100B as a marker (Table 1). Although the number of patients evaluated was small there was a suggestion that increasing CMC counts were associated with decreased patient survival [hazard ratio of 1.46, 95%, Confidence Interval (CI) (0.91-2.36), p = 0.12 after log-transformation of the counts]. An exploratory analysis within the patients with cutaneous melanoma, positive for *BRAF ^V600E^* mutation, (N = 7) identified a stronger trend between lower CMCs (≤ 100/mL of blood) and increased survival (p = 0.06). Further analysis did not identify any relationship to site of metastasis, or whether the patient was receiving systemic therapy at the time the sample was obtained.

### Disease specific miRs are enriched in Eluate fractions of metastatic melanoma blood samples

**Figure 5:**
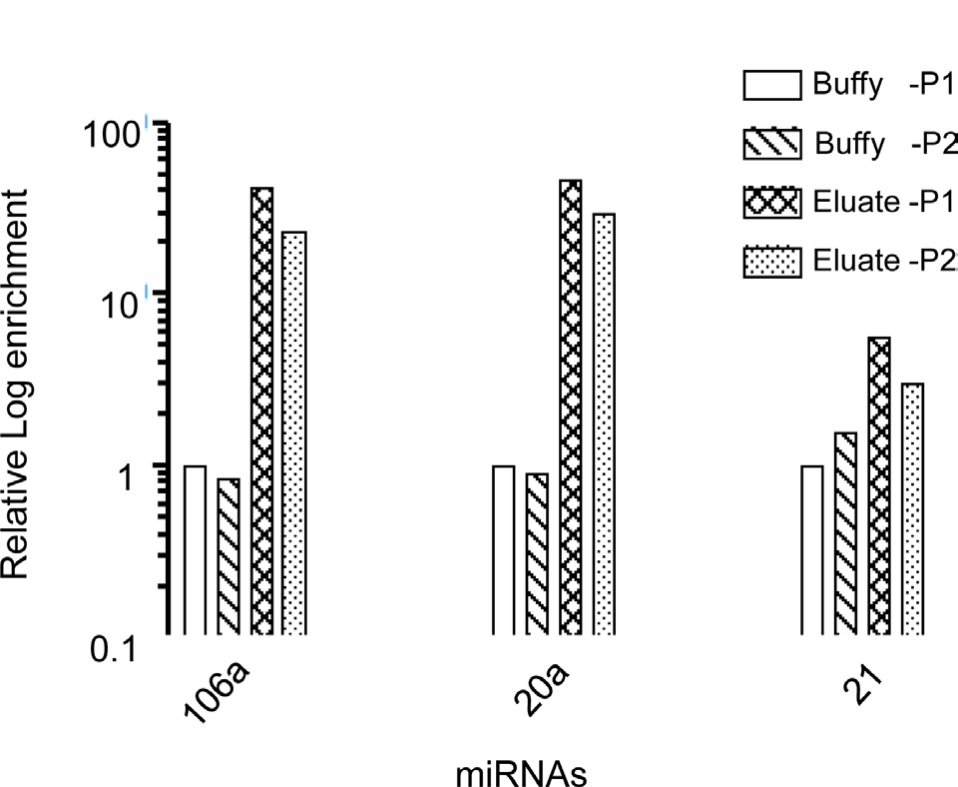
Melanoma specific miRs are enriched by the negative selection method miR profiles for metastatic melanoma samples pre- and post- separation by negative selection from 2 patients P1 and P2. Note that all the miRs are enriched in the post separation sample compared with pre-enrichment buffy coats, indicating substantial enrichment of CMCs shown in log scale.

The high CMC number isolated in the Eluate fraction without attachment of the targeting magnetic antibody allowed us to perform additional molecular analyses of the CMCs. The CMC negative selection method was thus further validated by assessing miRs in enriched fractions from two test patients. Eluates from two patients with malignant uveal melanoma were quantified for microRNA (miR-20a, 21, and 106a) [20], using real time qRT-PCR (Figure 5). miRs associated with uveal melanoma in the CMC enriched samples (Eluates) were increased in comparison with the original cell suspension (Feed).

## DISCUSSION

In an effort to develop a robust CMC enumeration procedure, a negative selection platform was combined with detection using selective intracellular melanoma markers and enumeration with a semi-automated imaging and analysis technique. Previously, the negative selection and enumeration of circulating tumors cells was shown to be effective in other types of malignancies (head and neck squamous cell carcinoma and breast cancer) [12-15]. However, a robust detection and enumeration had to be optimized for melanoma in addition to the negative separation process. The efficacy of this method was tested on simulated samples spiked with cultured melanoma cells and on 11 melanoma patients and 10 healthy donor blood samples. Results in spiking experiments with the SKMEL-28 melanoma cell line demonstrated high sensitivity and specificity of capture and detection of CMCs. Moreover, the negative selection capture method was validated by enrichment of melanoma specific miRNAs 20a, 21 and 106a [20] from enriched CMC samples. MiR-20a, 21 and 106a are shown to be increased in colorectal cancers [21-23].Circulating miR-21 is increased in breast cancers [24].

CMCs isolated from patient blood had heterogeneity in size, morphology and marker distribution. These differences may indicate underlying genomic heterogeneity and may play a role that may be clinically relevant in progression and evolution of the disease [25]. Like others, we identified cells that were simultaneously positive for both melanoma markers and CD45 [11, 26, 27]. The identity of these dual positive cells is unknown. Their presence could be due to artifacts of the co-immunostaining procedure or due to the presence of cells of hematopoietic origin capable of presenting the melanoma tumor antigens or hypothetical tumor-leukocyte hybrids.

Our results indicate a large range of CMC numbers between patients (21 to over 28,000/mL); in comparison, the healthy donor blood cohort had significantly lower counts or no cells (0-3 cells/mL) positive for CMC markers. These cells in normal blood samples expressing Melan-A or S100B proteins may be of neuroendocrine origin or artifacts of the antibody sandwich assay. The limited number of patients tested precludes a definitive assessment of the prognostic value of CMC counts. Nevertheless, preliminary analysis of the overall survival based on the CMC counts within our tested cohort of patients indicated a negative trend between survival and CMC counts in all patients (p = 0.12) and particularly for *BRAF^V600E^* cutaneous melanoma patients (p = 0.06). This observation should be viewed with caution, however, due to the small number of patients studied and the limited follow-up available for some patients. Nonetheless, the results are intriguing and point to the need for a larger, confirmatory study.

Associations have been established between EpCAM marker-positive CTC counts and overall survival in other malignancies [1, 3] and between CD146 melanoma-marker positive CMC counts and survival in melanoma [11, 26]. The past several years have seen dramatic advances in cell separation technologies, driven largely by the need for increased sensitivity and specificity in rare CTC detection [28, 29]. Early work based on fluorescence-activated cell sorting (FACS) analysis [27, 30] for CTCs was limited due to a requirement of high throughput for rare event detection [31, 32]. Even newer technologies for positive capture rely on differences in physical properties between the transformed and normal cells, such as those used for size filtration [33-35], label-free dielectric [36, 37] and magnetic [38, 39] separations. Advances in microscopy and high throughput imaging instrumentation made it possible to visualize and count CTCs directly in blood leukocyte smears [40, 41] and have raised issues of potential false negative results [42-45]. Overcoming challenges of targeting CTC using size, physical characteristics or a tumor specific marker has led to a better understanding of the mechanisms of tumor antigen deregulation and tumor cell heterogeneity [32]. At the same time it has stimulated a vigorous debate about CTC definition criteria and has motivated further research and development [46-50].

Investigations into the biological role of CTCs, their tumorigenic potential and contributions to disease progression have led to insights into the complexity of tumor progression. For example, apoptotic CTCs are not tumorigenic; however, their presence was found to be correlated with the involvement of liver as a specific site of metastasis in colorectal cancer patients [51]. Presence of CTCs in the cerebrospinal fluid (CSF) was correlated with the early detection of brain metastasis, and their numbers in the CSF correlated to treatment efficacy [6]. Alternate biomarkers such as ctDNA and mutant mtDNA are also being studied to gain insights into mechanisms of tumor progression and dynamics [8, 9]. Massive parallel sequencing of circulating nucleic acids are being explored for driver and secondary mutations that may pave a way toward personalized medicine. Additionally, analyzing CTCs at single cell genomic level may contribute to insights and understanding of non-driver mutations called “private” mutations, that could be clinically relevant due to the availability of target drugs [52].

In summary, this study elucidates the following advantages of CMC enrichment by negative selection, *viz*., un-biased CMC enrichment without a predetermined cell surface marker, robust, sensitive detection, and accessibility to molecular analysis. The detected trend of decreased survival with the increased CMC count warrants consideration of additional studies on a larger patient population. Additionally, negative selection offers opportunity for *in vitro* CMC culture or *in vivo* xenograft assays, since the CMCs are untouched and unmodified by immunomagnetic reagents. The feasibility of such assays has been demonstrated recently in the case of the metastatic breast and prostate cancer CTCs [47, 53]. Indeed, the prostate cancer study isolated CTCs using a “negative selection” protocol similar to ours [53]. In melanoma, successful xenografts of cells from patient tumors into non-obese diabetic, severe combined immunodeficient, IL-2 receptor-γ chain null (NSG) mice have demonstrated correlation with clinical progression of the disease [54]. It would be interesting to assess correlations between success in CMC xenografts and disease progression.

## MATERIALS AND METHODS

### Peripheral blood sample acquisition and pre-processing

Blood samples were obtained from patients with advanced metastatic melanoma after clinical evaluation and patient consent under an IRB approved protocol. Samples were collected in vacutainer tubes containing EDTA as anticoagulant. All samples were stored at 4°C, and processed within 24 hours of blood withdrawal (N = 11). Negative controls were similarly donated by apparently healthy adult volunteers under an IRB approved protocol (N = 10). The blood samples in the vacutainer tubes were centrifuged at 300 g in an Allegra CR centrifuge (Beckman Coulter, Inc.) for 10 minutes at 22° C. The plasma was carefully aspirated out and discarded after centrifugation. The buffy coat (~1 mL), a thin whitish band containing leukocytes and CMCs between the plasma and the RBC pellet, was aspirated gently to avoid contamination with RBCs and placed in a 50 mL tube. The contaminating RBCs were lysed using an isotonic ammonium chloride buffer (0.154 M ammonium chloride, 10 mM potassium hydrogen phosphate, 0.1 mM EDTA) for 5 minutes at room temperature (RT) and centrifuged at 4° C for 7 minutes at 250 g. The cells were washed with phosphate buffered saline (PBS) and resuspended in 1 mL of Separation Buffer (PBS, 2% FBS, 0.5 mM EDTA).

### Anti-CD45 antibody and immunomagnetic labeling

Twenty five μL of anti-CD45 antibody tetramer (MEM-28 Clone, CD45 Depletion kit, StemCell Technologies, Inc.) was added to the cells suspended in 1 mL of Separation Buffer and incubated for 30 minutes, at RT in a rotator. Subsequently, 50 μL of magnetic nanoparticles (Stem Cell Technologies, Inc.) was added, and the incubation continued for another 15 minutes. The volume was made up to 5.5 mL with Separation Buffer. This cell sample was referred to as “Feed”.

### Magnetic separation of the labeled white blood cell fraction

Five mL of Feed was loaded on an automated quadrupole magnetic cell sorter (Supplementary Figures S1 and S2). The remaining 0.5 mL of Feed was reserved for cell counts in initial experiments. The flow rate was set to 10 mL per minute. The labeled cell suspension was passed through the magnet in a “catch and release” mode. Most of the magnetically labeled CD45+ cells were retained inside the magnet (Retentate fraction) and non-magnetic cells were collected as Eluate fraction. As a part of the self-consistency check of the separation method, the cell number balance was performed by comparing the total number of cells in Feed to the sum of cells in Eluate and the Retentate fractions. The CD45+ cells retained on the magnet were collected by moving the separation channel out of the magnet and flushing at a high flow rate of 100 mL per minute (Retentate). This fraction was also tested for the presence of CD45+ cells and CMCs. Both Eluate and Retentate were centrifuged at 300 g for 10 minutes, and the cell pellets were re-suspended in 1mL of Separation Buffer for Eluate and 1.5 or 2 mL for Retentate (the latter to compensate for higher cell numbers in the Retentate as compared to Eluate).

### Cell count, fixation and Cytospin preparation

Fifty to a hundred μL of Feed, Eluate and Retentate aliquots were mixed with 450-400 μL of RBC lysis buffer (as above) and incubated for 10 minutes at RT for viability counts (Vi-CELL® Cell Viability Analyzer Beckman Coulter, Inc., Brea, CA). The appropriate cell size range and dilutions were selected, and the program was run to obtain the viable cell counts using Trypan Blue exclusion test. These counts were used to check total cell number mass balance before and after the separation. For cytospin preparations, the cell pellet was fixed in 1 mL of 2-4% of formaldehyde, for 20 minutes at RT to overnight at 4 °C and resuspended in the same volume of PBS and stored at 4 °C until staining. The volume required for 50,000 cells used for Cytospin preparations (Shandon Cytospin III centrifuge) was calculated based on the cell concentration in suspension (typically 25-50 μL).

### Immunocytochemistry

S100B and Melan-A were chosen as melanoma markers because of their ubiquitous presence in most melanomas [17]. These proteins are intracellular and have the following characteristics: S100B belongs to family of calcium binding proteins, such as Calmodulin and Troponin C. It is localized in the cytoplasm and sometimes in the nucleus. It is present in almost all benign naevi and malignant melanocytic tumors [17]. Melan-A/Mart1 (Melanoma Antigen/Melanoma Antigen Recognized by T cells) is a single pass, trans-membrane protein present in Endoplasmic Reticulum (ER), Golgi network, and vesicular membranes in the cytoplasm. The pan-leukocyte CD45 antibody was used to decrease the likelihood of CMC misclassification (targeted to a different epitope than the CD45 antibody used for the magnetic leukocyte depletion). Anti S100B and Melan-A antibodies were purchased from Novus Biologicals (Littleton, CO), and anti-CD45 antibodies from Dako (2B11 +PD7/26 clones, Carpinteria, CA) and Molecular Probes (HI-30 clone, Life Technologies, Carlsbad, CA). These antibodies were used at a dilution of 1:50 and 1:100, respectively. Secondary antibodies tagged to Alexa fluorophores (Alexa 488 and Alexa 568) were purchased from Molecular Probes (Carlsbad, CA). These were used at a concentration of 1:500 and 1:2,000 respectively. The cells on the Cytospin slides were permeabilized with 0.1% Triton™ X-100 (Sigma Aldrich, St Louis, MO) for 10 minutes, washed with PBS, and blocked using a buffer containing 0.5% BSA, 1% human serum in PBS, for 1 hour at RT in the humid chamber. The primary and secondary antibodies were diluted independently in 0.25% BSA, 0.5% human serum in PBS and incubated sequentially for 1 hour and 45 minutes, respectively. The slides were washed 2 times, for 10 minutes each, in PBS containing 0.01% Tween® 20 (Sigma Aldrich, St Louis, MO) after each step of incubation. At the end of the last wash step, a coverslip was placed on each slide over a drop of Vectashield® hard set mounting media (Vector Laboratories, Inc., Burlingame, CA) containing DAPI for nuclear staining. The slides were imaged after the Vectashield medium was set (12-24 hours post-mounting).

### Microscopy image acquisition

The entire monolayer cell deposit on the cytospin slide (Area of Interest or AOI) was imaged with a 20× objective (Supplementary Figure S3). A Leica DMR series fluorescence microscope (Leica Microsystems, GmbH) fitted with a motorized stage and interfaced with ImagePro acquisition and analysis software (Media Cybernetics, Rockville, MD) was used. The entire AOI was scanned using three band-pass excitation and emission filters corresponding to DAPI, Alexa 568 and Alexa 488 channels. The images were captured using a Retiga 4000R CCD camera with monochromatic sensors (QImaging, Surrey, BC, Canada) using a virtual rectangular reference grid of high magnification (200×) Field of Views (FOVs) and an automated focus adjustment in all three channels. The FOV grid covered the entire AOI on the cytospin slide and typically consisted of over 300 FOVs. These images were tiled and stitched as a single large image (Supplementary Figure S4), or alternatively, a sequence of FOVs from each channel was saved as a stack for easy analysis. The images in three channels were merged for viewing and presentation.

### Computer-assisted image analysis

The image analysis was done by two different methods for comparison of accuracy and ease-of-use. 1) Manual counts were performed using ImageJ. The pixel intensity thresholds, on a scale of 0-255, were set using the default algorithm in the ImageJ, and by adjusting the slider until the background pixels were equal to zero in each channel. The correlated images for nuclei in DAPI channel (blue), leukocytes with CD45 channel (Red) and Melan-A or S100B channel for melanoma CMCs (green) were merged to obtain a RGB image. To be counted as a CMC, a cell must have Green pixels (from Melan-A or S100B), no Red pixels (from CD45) and a DAPI stained blue nucleus (Figure 1). Criteria from Supplementary Table S1 were applied here as well in order to minimize false positive counts. 2) In addition, an automated script for ImagePro® was used. A customized image processing algorithm coded for ImagePro® automated the counting process of all images (ImageIQ, Inc., Cleveland, OH). For these analyses, all images acquired in individual channels were stored as sequences. The sequence of DAPI images were first analyzed using spectral intensity filtering and segmentation followed by morphological filtering based on aspect ratio and circularity criteria to identify the nuclei. “Nuclear masks” were created using the binary rendition of these images. Based on the cell fluorescence intensity scatterplots for CD45 (Red) and Melan-A or S100B (green) channels, global thresholds were selected by the user (Supplementary Figure S7) and applied to these channels. The algorithm selected the “Green and not Red” cells as putative CMCs after the Green and Red channels were merged with the masks of the blue channel. The algorithm tabulated in a Microsoft Excel sheet providing counts for the entire AOI in four object classes: green (putative CMCs), red (leukocytes), yellow (red and green) and null (not green and not red). The images were reviewed manually at the end of each analysis to eliminate any false positives from the counts based on the criteria listed in Supplementary Table S1.

The effect of pixel intensity threshold selection on an image of a patient blood sample is illustrated in Supplementary Figure S5. The negative control for healthy volunteer blood is shown in Supplementary Figure S6. CMCs identified according to the criteria listed in Supplementary Table S1, are marked with green arrows. Leukocytes are marked with red arrows. Clusters of fluorescence that did not appear to have a clear morphology of a cell and associate with a nucleus were considered as debris. For some patients (but not for healthy controls) CMC enumeration was confounded by high pixel intensity background, a likely artifact of the dual antibody sandwich assay used for cell staining. Therefore, the CMC count was performed with a bias against the green signal (CMC marker) for the patient samples and bias toward green signal for the healthy controls for greater stringency as illustrated in the scatter plots showing signal thresholds (Supplementary Figure S7).

### CMC enumeration (normalized to 1 mL whole blood) and error analysis

CMCs were enumerated based on the highest amount of antigen expressed, either Melan-A or S100B. The CMC number concentration per 1 mL of blood, *nCMC*, was calculated by extrapolation of the CMC counts from the cytospin slide by image analysis (as described above) taking into account the ratio of the cytospin sampling volume to the total blood volume used for the analysis. An example of the calculation and the associated sampling error analysis is shown in the Supplementary Information. Patient data were summarized using medians and ranges. Overall survival was measured from the date the blood sample was obtained to the date of death or last follow-up. As exploratory studies, associations between *nCMC* and patient demographics and overall survival were examined using Mann-Whitney and Log Rank tests, proportional hazards models, and Spearman correlations. The Mann-Whitney was also used to compare *nCMC* between melanoma patients and healthy volunteers. All tests of statistical significance were two-sided and performed using SAS version 9.2 (SAS Institute, Inc, Cary, NC), StatXact version 9 (Cytel Software Corp., Cambridge, MA) or Graphpad Prism® (GraphPad Software Inc., La Jolla, CA).

### Real-time quantitative Polymerase Chain Reaction (qPCR) analysis for disease specific microRNA (miR) enrichment

Samples from two patients were processed using the same protocol as described above and analyzed for specific miRs from the enriched CMCs. Briefly, RNA was isolated from the pre-enriched buffy coat samples (Feed), and post-enriched Eluate fractions of the patient blood stored in Trizol. Equal amounts of RNAs were used with specific primers for the miRs. The quantification reported is relative to the original cell suspension (Feed). The cells were lysed, and lysates were applied to the AllPrep DNA/RNA/Protein Mini Kit (Qiagen Inc., Venlo, Netherlands), which enables the isolation of DNA, large RNA fragments (mRNA >200 bp), small RNA fragments (miR and noncoding RNA <200 bp), and proteins in four separate aliquots from as little as one cell. To address miRs expressed by leukocytes present, an aliquot was also analyzed by qRT-PCR to assess Melan-A and CD45 signals, to estimate the ratio of the tumor cell-specific signal over the leukocyte-derived signal. Each qRT-PCR assay was done at least twice with marker-positive, marker-negative, and reagent controls. Tissue processing, RNA extraction, qRT-PCR setup, and post-qRT-PCR product analysis are carried out in separate areas to prevent cross contamination. Until consensus has been established on a robust reference miR set, normalizing on the mean expression of all expressed miRs in both groups was considered the optimal method when multiple miR transcripts are measured at the same time. U6 SnRNA was used as a standard control RNA for these experiments.

## SUPPLEMENTARY FIGURES, TABLES, INFORMATION






