# Supplementary material for: Enrichment of circulating melanoma cells (CMCs) using negative selection from patients with metastatic melanoma
Source: Oncotarget. 2014 Feb 3;5(9):2450–61. doi: 10.18632/oncotarget.1683 (PMC4058018; doi:10.18632/oncotarget.1683)
Supplement: Supplementary file 1 [file oncotarget-05-2450-s001.pdf]

Enrichment of circulating melanoma cells (CMCs) using negative selection from patients with metastatic melanoma- Joshi et al

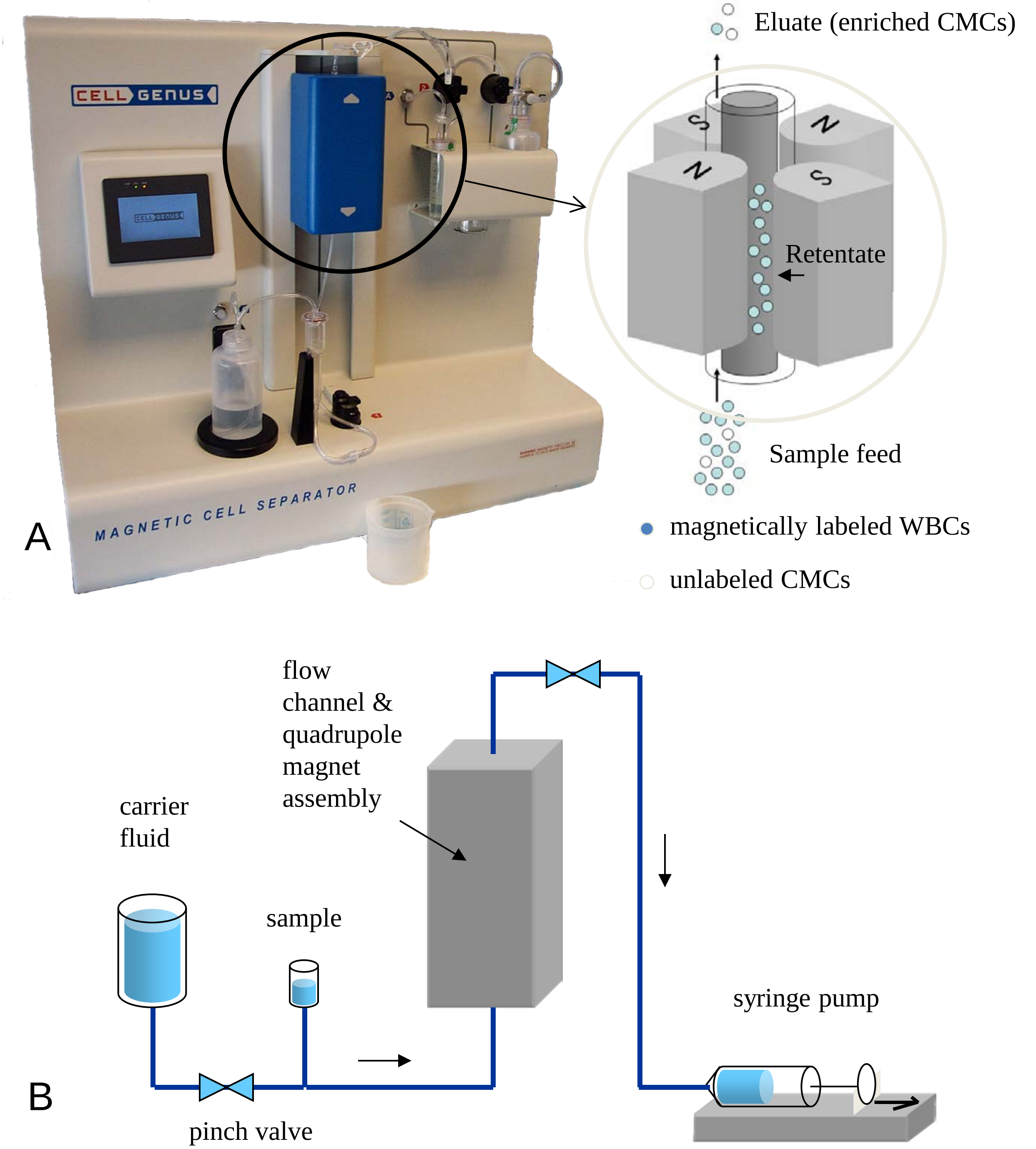

Supplementary Figure S1: Principle of operation of the magnetic flow sorter in application to negative selection of CTCs. (A) Schematic illustration of the annular flow channel placed in the aperture of a quadrupole magnet (N-S-N-S) and its location relative to other system components (photograph). Competing forces of radial magnetic forces and axial flow viscous shear acting on a cell lead to separation of the unlabeled CTCs (white circles) in the Eluate stream and retention of the magnetically labeled WBCs inside the magnet (blue circles). (B) Schematic of the fluidic system. Pinch valves provide means to replace syringes on the syringe pump for sequential filling, fraction collection (Eluate and Retentate) and flushing of the system.

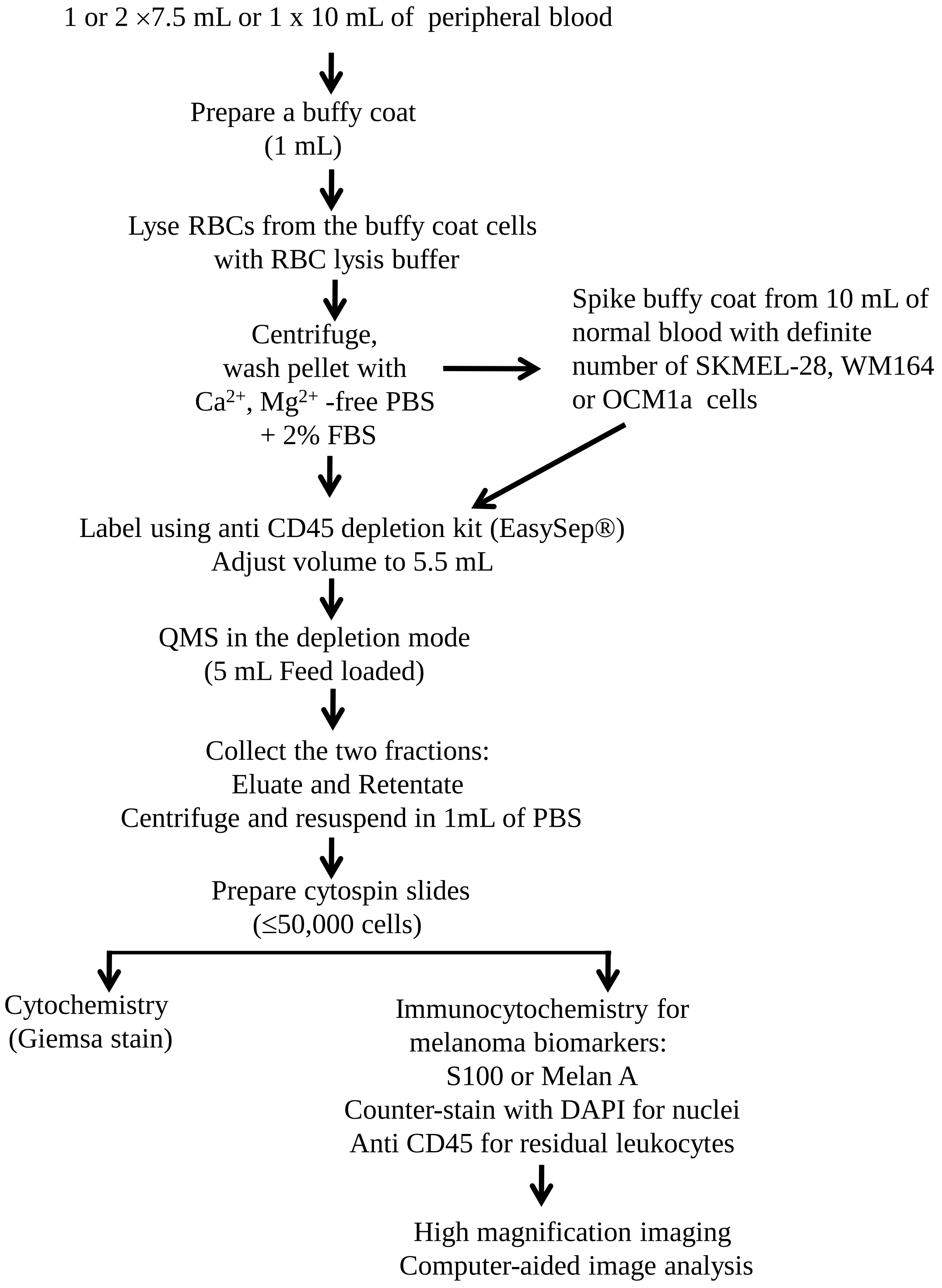

Supplementary Figure S2: Flow chart for negative enrichment. Flow chart depicting the protocol of separation, detection, imaging and analysis as applied to blood samples donated by metastatic melanoma patients. Spike sample runs were done similarly except the cultured melanoma cells from cell lines were added to the buffy coat at step 4 as shown in the figure in order to exclusively assess the quadrupole magnetic separator performance.

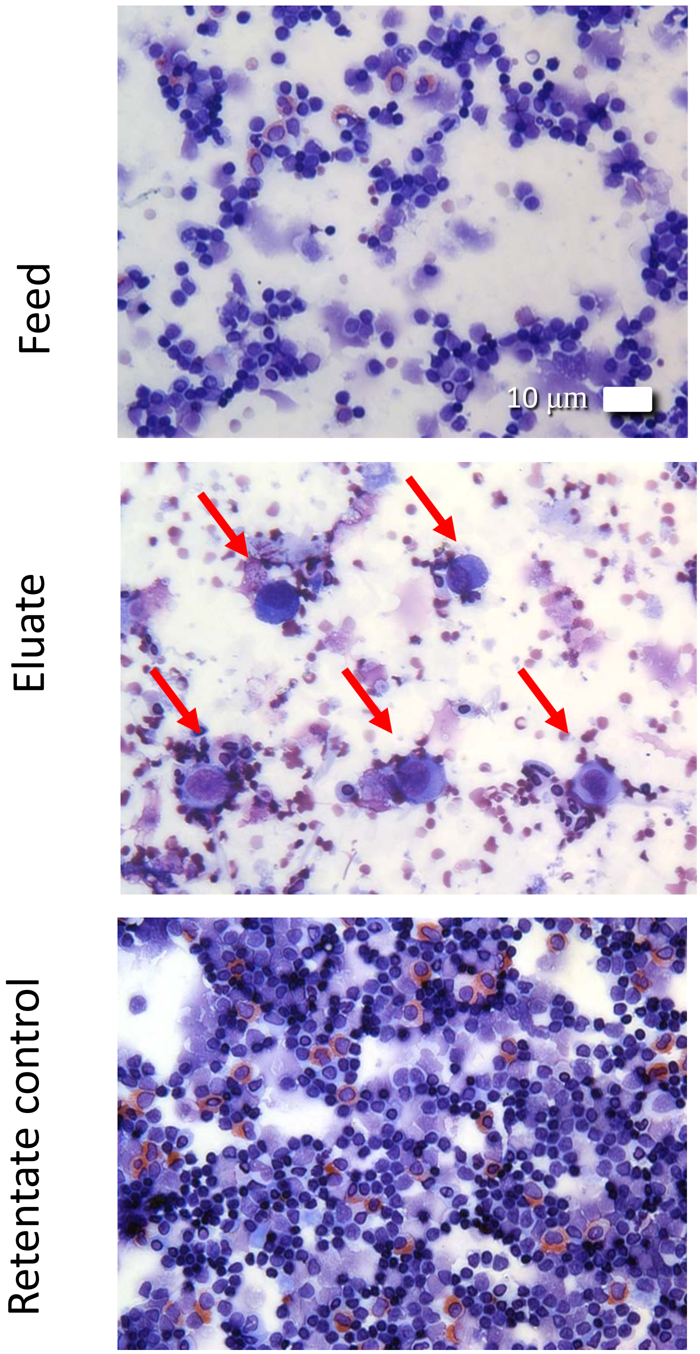

Supplementary Figure S3: Enrichment of ten OCM1a cells spiked directly in 10 mL of normal blood. The specificity of the negative separation tested by spiking OCM1a cell line into whole blood, at 100 cells per 10 mL of blood, labeled against CD45 for leukocyte removal, separated on quadrupole magnetic separator and subsequently analyzed by cytochemistry. The large OCM1a cells are clearly visible in the enriched Eluate fraction. A small amount of leukocytes are also seen in the OCM1a enriched Eluate fraction. Most leukocytes are captured in the Retentate fraction.

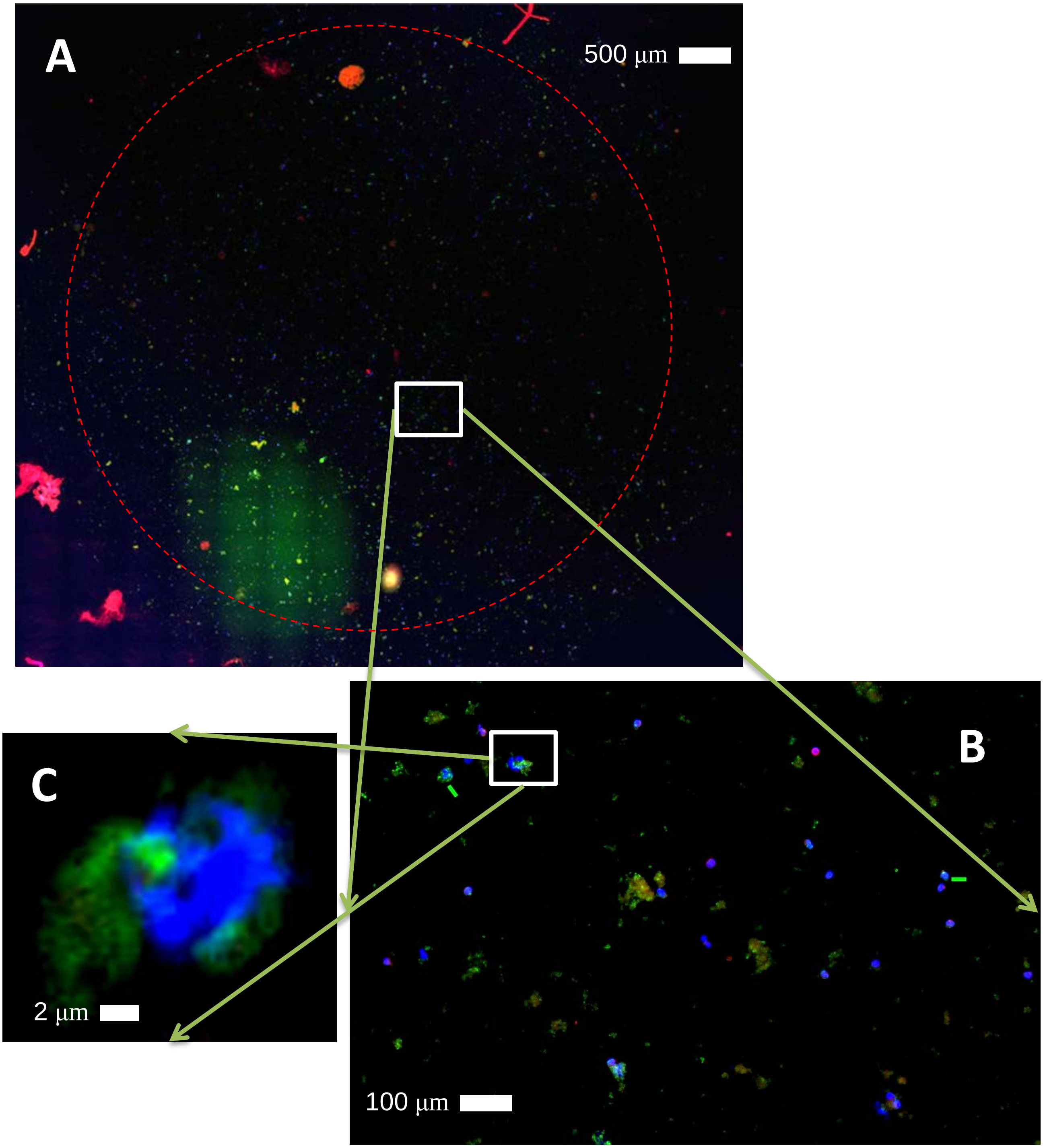

Supplementary Figure S4: Example for image acquisition and processing. The image (A) is a montage of 17×17 fields of view (FOVs) using 20× magnification objective that covers the entire area of the cytospin slide ROI (indicated by a red circle). One FOV is shown in (B). The cells can be digitally zoomed in as illustrated in C. Typically ~50,000 total cells were counted per slide, equivalent to a blood volume of 5 – 25 µL. Key to colors: Blue - DAPI (cell nuclei), Red – CD45 (leukocytes), Green –S100B or Melan-A

A

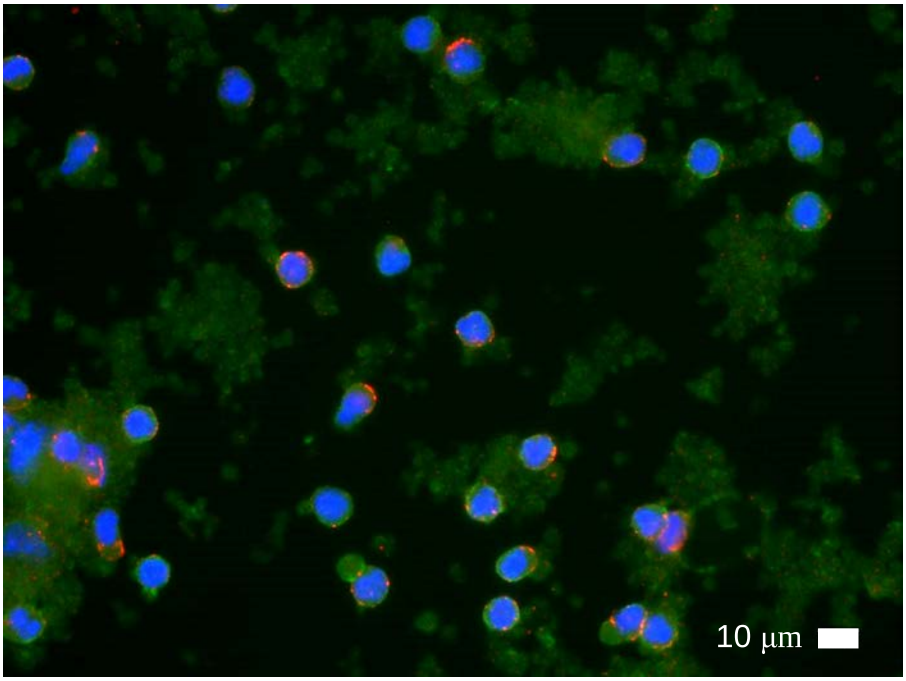

B

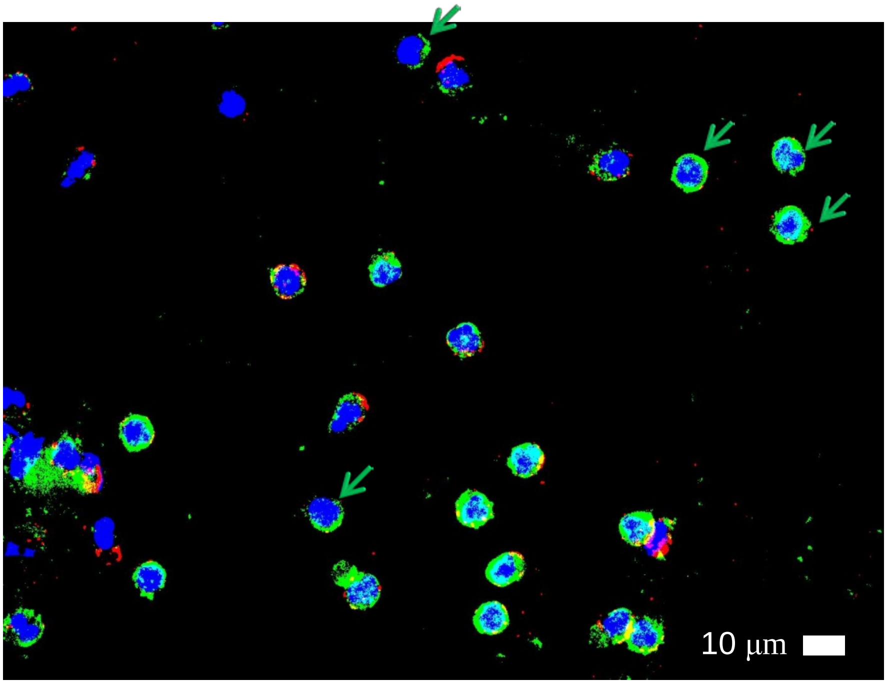

Supplementary Figure S5: Image analysis method. An example of image analysis performed on one of Eluate fractions of patient blood sample stained with S100B and CD45. (A) original RBG composite image; (B) same image after the application of thresholds. (Red at 25, Green at 80 and Blue at 255). Blue - DAPI (cell nuclei), Red – CD45 (leukocytes), Green –S100B Here total # Cells = 32, # of Green = 5, # of Dual staining = 26, # Unstained cells = 1 Scale bar = 10 μm.

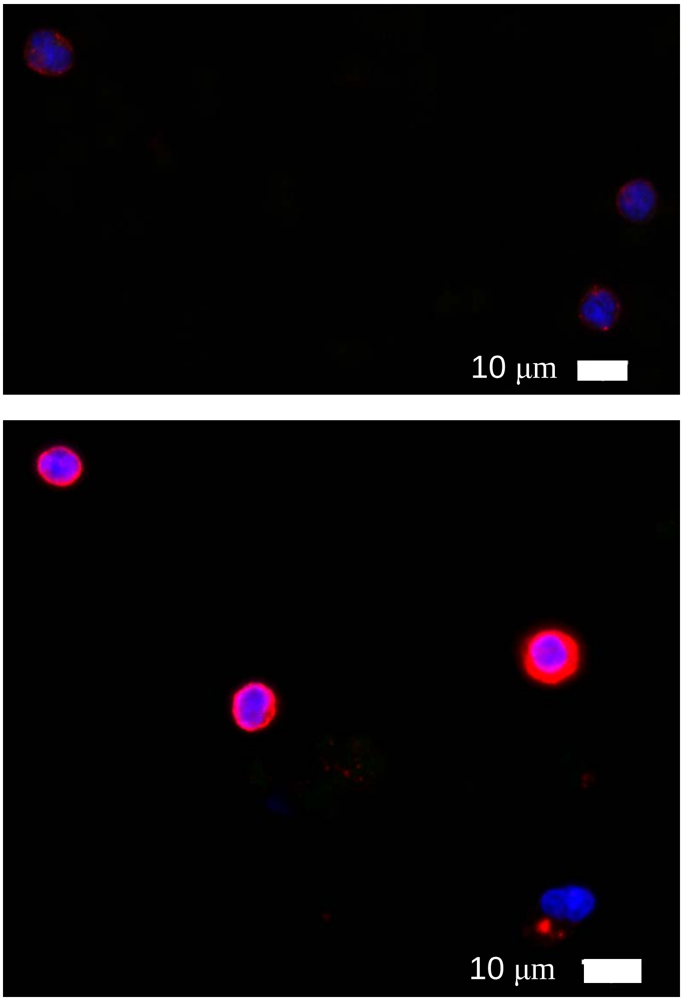

A

B

Supplementary Figure S6*: Images for co-immunostaining with CD45 and Melan-A/S100 on healthy blood cells.* The healthy donor blood negative controls show no presence of CMC-like objects by the same immunocytochemical and morphological criteria as used in previous in these images. Figures. Key to colors: Blue - DAPI (cell nuclei), Red – CD45-PE (leukocytes), Green – Melan-A (A) or S100B (B). Slide scan for 10samples done for each marker resulted in a maximum of 3 cells that met the definition of CMCs. Scale bar = 10 μm.

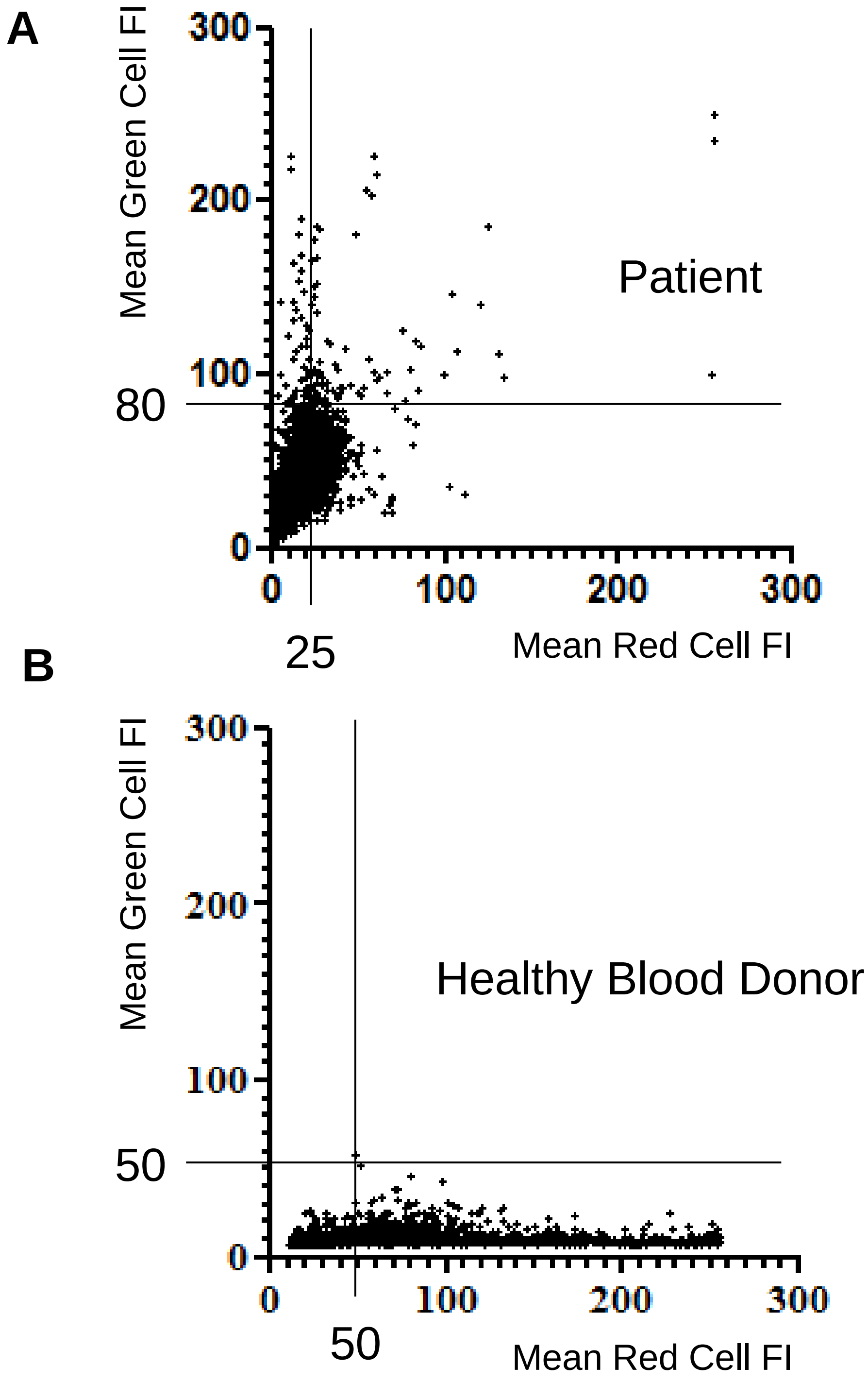

Supplementary Figure S7: **Selection of threshold for image analysis.** Example of threshold cut off values selection using the cell fluorescence intensity (FI) scatter plots for Melan-A (Green) and CD45 (Red). All points that are associated with an authentic nucleus (DAPI fluorescence) and that fall in the top left quadrant add up to the number of CMCs. All images were manually reviewed after the custom scripts for ImagePro listed numbers for each color combination (Red+Green-, Red-Green+, Red+Green+ and Red-Green-). (A) Patient 7. The threshold gates are set at: 25 for Red and 80 for Green. (B) Healthy donor control (Donor 5). Here the threshold gates are set at 50 for Red and 50 for Green resulting in 1 CMC (a conservative estimate).
