# Supplementary material for: Enrichment of circulating melanoma cells (CMCs) using negative selection from patients with metastatic melanoma
Source: Oncotarget. 2014 Feb 3;5(9):2450–61. doi: 10.18632/oncotarget.1683 (PMC4058018; doi:10.18632/oncotarget.1683)
Supplement: Supplementary file 2 [file oncotarget-05-2450-s002.pdf]

Enrichment of circulating melanoma cells (CMCs) using negative selection from patients with metastatic melanoma – Joshi et al

Supplementary Table S1: Selection criteria for CMC enumeration.
These criteria are applied to images that were processed using pixel luminosity range analysis (as described in text).

**Parameter**	**Clear Nuclear morphology**	**Fluorescence pattern**			**Debris**	**Clustering**
		**S100B**	**Melan-A**	**CD45**		
**CMC**	Present	Cytoplasmic, nuclear; amorphous	Cytoplasmic; amorphous or punctate	Low or Absent	Absent	Absent
**Leukocyte**	Present	Low or absent	Low or Absent	Present	Absent	Absent
**Dual positive**	Present	Present	Present	Present	Absent	Absent

Supplementary Table S2: **Number of Melan-A and S100B positive cells detected in healthy blood.** The healthy donor blood controls for the test of Melan-A and S100B positive cell enumeration. The true identity of these cells is unknown. A small amount of non-specificity of the staining pattern is likely due to the use of secondary antibodies and image analysis technique used. A median equal to the highest value of 3 cells /mL of blood was selected as the measure of non-specific Melan-A and S100B binding (both categories combined in the statistics, N of donors = 10). N/D – not determined

**Sample Number**	**nCMC/mL blood by antibody marker**	
	**Melan-A**	**S100B**
1	0	N/D
2	N/D	1
3	3	N/D
4	0	3
5	0	3
6	3	3
7	3	3
8	3	0
9	0	0
10	3	N/D
