# Supplementary material for: Enrichment of circulating melanoma cells (CMCs) using negative selection from patients with metastatic melanoma
Source: Oncotarget. 2014 Feb 3;5(9):2450–61. doi: 10.18632/oncotarget.1683 (PMC4058018; doi:10.18632/oncotarget.1683)
Supplement: Supplementary file 3 [file oncotarget-05-2450-s003.pdf]

Enrichment of circulating melanoma cells (CMCs) using negative selection from patients with metastatic melanoma – Joshi et al

SUPPLEMENTARY INFORMATION

Selection of optimal flow rates on the quadrupole magnetic flow sorter

The quadrupole magnetic flow cell separator is described in detail elsewhere [10, 11]. Briefly, it is a flow sorter with a cylindrical channel placed inside a quadrupole magnet (Supplementary Figure S1A). The channel is connected to a sample vial and a buffer reservoir at the inlet and a collection tube or syringe at the outlet (Supplementary Figure S1B). The system was calibrated using mixtures of non-magnetic polystyrene microsphere standards (9.9 μm mean diameter, Duke Scientific Corp. Palo Alto, CA) and custom-made magnetic microspheres (6.2 μm mean diameter, Micromod Partikeltechnologie GmbH) of a magnetization similar to that of leukocytes labeled immunomagnetically with anti CD45 magnetic labeling reagent (EasySep®, StemCell Technologies Inc.). The mixtures were used for selecting optimal flow rates for maximum recovery of 9.9 μm microspheres in non magnetic fraction (Eluate) and 6.2 μm magnetic particles in the magnetic fraction (Retentate) based on the microsphere mass balance before and after the separation. The microsphere counts in the original and separated fractions were performed using an automated particle counter based on size differences between the non-magnetic and magnetic microspheres (Z2 Coulter Counter, Beckman-Coulter). The criterion of an acceptable negative separation was 10% or less contamination of the non-magnetic microspheres (prototypical CMCs) by the magnetic ones (prototypical leukocytes) in the Eluate fraction. The criterion was met in repeated experiments for the total flow rate of the microparticle suspension of 10 mL per minute (data not shown) which was subsequently selected as the flow rate for the ensuing cell separation experiments.

Analyzing capture efficiencies of CMCs using donor blood spiked with cultured melanoma cells

The protocol followed for peripheral blood and spiked cells separation is outlined in the flow chart in Supplementary Figure S2. Spiked cell experiments were done using WM164, OCM1a or SKMEL-28 cultured cells at specific numbers as surrogate CMCs. Forty five to 655 SKMEL-28 cells were spiked into buffy coat isolated from 10 mL of healthy donor human blood in order to obtain recovery efficiencies. The spiked cell suspension was labeled for leukocytes with anti-CD45 antibody and magnetic nanoparticles and sorted on the magnetic flow sorter. The Retentate and Feed fractions were collected and analyzed to determine spiked cell recovery (relative to Feed) and purity in each fraction. The cells were stained for Melan-A and CD45 to identify spiked SKMEL-28 cells and leukocytes for counting (Supplementary Figures S3 to S6). The SKMEL cell line spiking experiments were replicated for reproducibility. The statistical analysis included mean and standard deviation, confidence limit, and correlation analysis by linear regression.

Pixel luminosity threshold selection used for CMC classification

The pixel luminosity (on the scale of 0 to 255) of the two fluorochromes used to detect Melan-A or S100B (green) and CD45 (red) were plotted against each other as a scatterplot (Supplementary Figure S7). The intensities split into two distinct subsets whose centers of mass (or centroids) were located in different quadrants of 255×255 red by green pixel luminosity space. Quadrants were defined by color luminosity threshold values, typically in the range of 25-80 for both red and green channels based on a particular color scatter pattern characteristic of the blood sample. The threshold values were used to classify the object as a CMC (quadrant 2: high green, low red) or a contaminating leukocyte (quadrant 4: low green, high red). The objects in quadrants 1 and 3 were considered artifacts and were not counted. Examples of healthy donor blood (Supplementary Figures S7B) and advanced stage metastatic melanoma blood (Supplementary Figures S7A) samples illustrate differences in red v. green color scatterplots. In particular, the healthy donor samples were clearly discriminated from the patient samples by low intensity green values.

CMC enumeration

The CMC number concentration per 1 mL of blood, *nCMC*, was calculated by extrapolation of the CMC counts, *n*, in the cytospin sampling volume, $V_{cytospin}$ (mL) to the Eluate fraction volume, V_{Eluate} (mL) and normalizing the counts by the total blood volume, V_{blood} (mL) used for the analysis. An example of the calculation is best reviewed when inspecting the flow chart in Figure 2. The particular numerical values of various quantities listed below are used for the illustration only.

$$nCMC = n\frac{V_{Eluate}}{V_{cytospin}V_{blood}} \quad \text{per mL blood}$$

For instance, assuming typical values used in this study: V_{blood} = 15 mL, V_{Eluate} = 1,000 µL, $V_{cytospin}$ = 70 µL and n = 150, one obtains *nCMC* = 143 /mL.

Error analysis

The *nCMC* variance is calculated from the error propagation formula as applied to the expression for *nCMC*, above, and the rare event sampling error from Poisson distribution. Consequently, the *nCMC* relative standard deviation squared, CV^2_{nCMC}, is the sum of squares of relative standard deviations of quantities entering the above formula for *nCMC*, or:

$$CV^2_{nCMC} = CV^2_n + CV^2_{Eluate} + CV^2_{cytospin} + CV^2_{blood} \approx CV^2_n$$

The last approximation reflects the fact that the main contribution to *nCMC* variance is the variance of the CMC count on the cytospin slide, CV_n (considering that the all the volume variables can be controlled to better than 5% of their mean value). For Poisson distribution, the variance equals the expected value of repeated sampling (the mean). Assuming that $nCMC_{slide}$ = 150 in the example shown above is the measure of the most likely count (the mode of the distribution), one obtains:

$$CV_{nCMC} \times 100\% = CV_n \times 100\% = \frac{\sqrt{150}}{150} \times 100\% \approx 8\%$$

In absolute terms for the example shown above, *nCMC* = 150 ±12 /mL.

Limit of detection

In the example shown above, 15 mL blood volume is reduced to 1 mL of the negatively separated cell fraction (the Eluate) containing all the CMCs that originated from the initial blood sample (assuming no CMC losses during the separation process). An aliquot of that fraction (70 µL out of 1 mL Eluate) is taken for the cytospin analysis by immunocytochemistry and microscopy enumeration, which equals to approximately 1/15 fraction of the Eluate, and therefore 1/15 fraction all the CMC originating from the initial blood sample. In other words, the cytospin count corresponded to the number of CMCs in 1/15 fraction of the initial 15 mL blood volume, thus to a minimum of CMC count of 1 in 1 mL blood, which represents the theoretical limit of detection (LOD) of the current protocol (assuming no CMC losses during the separation process). The experimental LOD determined from cell spiking experiments, described above and in the main text, was higher than that, at 10 CMC/mL. If lower (better) LOD is required, additional 70 µL aliquots are available from 1 mL Eluate volume, up to the entire Eluate volume, corresponding to CMC count in the entire 15 mL blood sample. In that case, the theoretical LOD = 1 CMC/15 mL blood (assuming no CMC losses during the separation process). Although such LOD could be better than the current standard of 1 CTC/7.5 mL blood (by CTC CellSearch™) it would require high-magnification microscopy analysis of 15 cytospin smears, which was too time-consuming to be realistic at the time of this study. The current effort is directed towards improving the LOD to within the theoretical limit of 1 CMC/15 mL blood by using commercial automated slide image acquisition system and further improvements of the computerized image analysis algorithm.
